# A Comparative Study of MTA Solubility in Various Media

**Published:** 2011-02-15

**Authors:** Mohammad Ali Saghiri, Jack Ricci, Morteza Daliri Joupari, Mohammad Aeinehchi, Kamran Ahmadi, Niloofar Bahramian

**Affiliations:** 1. Department of Dental Material, School of Dentistry, Islamic Azad University of Medical sciences, Tehran, Iran.; 2. Department of Biomaterials and Biomimetics Director, New York University College of Dentistry, NY, USA.; 3. National Institute for Genetic Engineering and Biotechnology, Karaj, Iran.; 4. Department of Endodontic, School of Dentistry, Islamic Azad University of Medical sciences, Tehran, Iran.; 5. Material and Energy Research Center (MERC), Karaj, Iran.; 6. Department of Biomedical Engineering, Science and reach Branch, Islamic Azad University of Medical sciences, Tehran, Iran.

**Keywords:** Media, Mineral Trioxide Aggregate, Solubility

## Abstract

**INTRODUCTION:**

Solubility of root filling materials is heavily influenced by the environment they are in contact with. This study compared the solubility of ProRoot MTA in deionized water and synthetic tissue fluid.

**MATERIALS AND METHODS:**

Forty specimens of prepared MTA were immersed in deionized water and synthetic tissue fluid (20 samples each). The solubility was assessed after 7 and 28 days. Scanning electron microscope observation was also performed. The mean weight loss was evaluated using a digital scale. Data were analyzed using one-way ANOVA. Tukey test was performed for multiple comparisons.

**RESULTS:**

MTA solubility in synthetic tissue fluid was significantly lower than deionized water after 7 and 28 days (P<0.05). Secondary electron detectors revealed the presence of lumps and platelets on the surfaces of both specimens. Also, more voids were observed in specimen stored in deionized water.

**CONCLUSION:**

MTA dissolved faster in deionized water than synthetic tissue fluid. Despite this, the solubility of this material in both media was acceptable.

## INTRODUCTION

Dental materials including root-end fillings are subjected to various acids, enzymes, and fluids in the natural oral environment; therefore, their solubility or insolubility is an essential characteristic. Root-end filling materials cannot provide good sealing unless they are insoluble in periapical tissue fluids [1][2]. Using a material with low solubility in saliva and natural body fluids is critical in endodontics. Root-end filling materials must be insoluble in periradicular fluids; otherwise, delayed leakage and subsequent treatment failure will occur [3].

Mineral trioxide aggregate (MTA) has shown great potential as the material of choice for endodontic root-end filling. It has good sealing ability, low bacterial leakage, good marginal integrity and biocompatibility [2]. Therefore, MTA is the material of choice in retrograde treatments of teeth with open apices (apexification) [4]. MTA is mainly composed of Portland cement and bismuth oxide [5]. This issue has generated interests in the evaluation of Portland cement as an alternative to MTA. Danesh et al. claimed that MTA displayed superior properties and lower solubility than Portland cement [6]. On the other hand, another study reported that the solubility of white MTA was significantly greater than ordinary Portland cement [5][7]. Poggio et al. evaluated the solubility of IRM, Super seal, and Pro Root as root-end filling materials [8]. They concluded that they are virtually insoluble. Also, it is reported that the addition of CaCl2 to white MTA and white Portland cement decreased their solubility [9]. Furthermore, the degree of solubility rises as the water to powder ratio increases [10].The environment of a material will influence its degree of solubility and therefore this study aimed to compare the solubility of ProRoot MTA when stored in deionized water and synthetic tissue fluid.

## MATERIALS AND METHODS

This experimental study was conducted according to the International Organization by standardization guidelines (ISO 6876:2002) for evaluating dental root canal sealing materials [11].

Twenty sachets of ProRoot MTA (Tooth- colored, Dentsply, Tulsa Dental, Tulsa, Ok, USA) were mixed with their accompanying ampoule according to the manufacturer's instruction and inserted into 40 stainless steel (SS) ring molds (internal diameter: 20±1mm, height: 1.5±0.1mm). Flat glass plates having dimensions larger than the maximum dimension of the ring molds were used to flatten the surface of the specimens. The face of the glass tubes was pressed against the specimens and carefully removed to leave a flat and uniform surface. Cement flushes were removed carefully. All specimens were placed in a 37ºC incubator with not less than 95% humidity for a time period 50% longer than the setting time stated by the manufacturer. Each ring was weighed twice by means of a digital scalar with 0.1mg accuracy (Mettler College, Germany) before and after filling. They were randomly divided into two groups of 20 each and were stored in deionized water and synthetic tissue fluid (STF) using micro fence. The STF was a phosphate buffer saline solution (pH=7.2) with the following composition: 1.7g KH2PO4, 11.8 g Na2HPO4, 80.0g NaCl, and 2.0g KCl in 10L of H2O. Specimens were immersed in a container with 100cc fluid and the container was sealed by one Parafilm stripe (Parafilm "M", Laboratory Film, Chicago) and placed in incubator (37ºC and no humidity). Ten specimens from each group (20 total) were taken out from incubator after 7 days and again another set (total=20) taken out after 28 days; they were washed with distilled water, dried with drier paper, placed in incubator (37ºC with no humidity) for 24 hours and weighed again. Solubility was verified by the amount of weight loss.

Also, one specimen from each group was selected and prepared for scanning electron microscopy to observe the surface characteristics and effects of STF and deionized water on the MTA surface. The surface was sputter-coated and observed under scanning electron microscope (SEM) (Leo 440i; Oxford Microscopy, Oxford, UK) using secondary electron mode (×500 and ×2500).

One-way ANOVA analysis was used to compare the medias’ cumulative data. Statistical significance was set at α=0.05. Tukey test was performed for multiple comparisons.

## RESULTS

**Figure 1:**
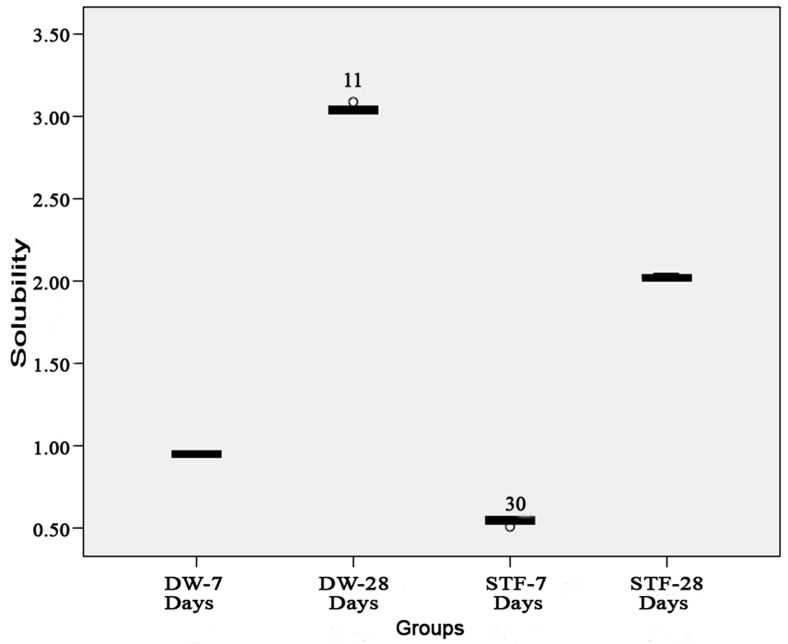
Box plots of solubility values in each group stored in deionized water and synthetic tissue fluid. The mean±standard deviation values were 0.95±0.00, 3.04±0.02, 0.54±0.01 and 2.02±0.01 after 7 and 28 days in deionized water and STF, respectively.

**Figure 2:**
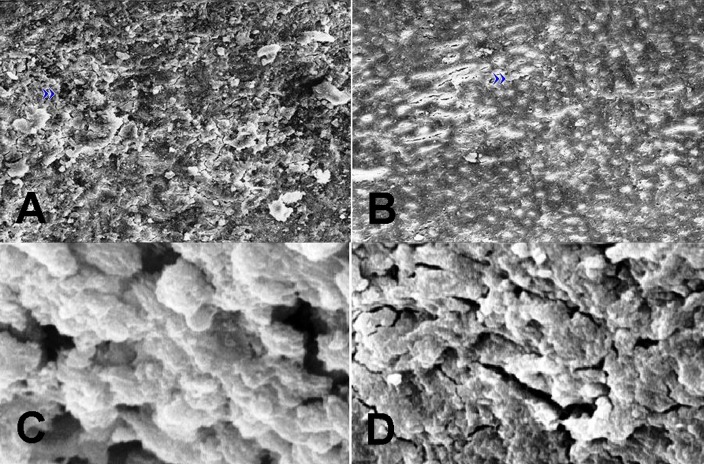
SEM micrographs of specimens stored in synthetic tissue fluid (a,c) and deionized water (b,d). More voids (») can be seen on the surface of MTA stored at deionized water after 28 days. (Original magnification ×500 and ×2500)

The solubility of MTA in deionized water and synthetic tissue fluid after 7 and 28 days are illustrated in Figure 1. Tukey test revealed significant difference between groups (P<0.05). SEM micrographs (Figure 2) revealed little porosity and predominantly uniform geometrical shaped crystals which covered the surfaces of all specimens at 28 days; however, large porosities were found on the surface of the specimens stored in deionized water.

## DISCUSSION

A suitable root-end filling material must provide an apical seal that inhibits the leakage of irritants from the root canal system into the periradicular tissues [1][12]. Insolubility is a desirable property for restorative materials in dentistry; moreover, it is also an ideal characteristic for root-end filling materials [13]. Although in clinical situations only part of the root-end fillings are in direct contact with the aqueous environment (i.e. periapical tissues) [10], under the conditions of the present study the whole specimen was in contact with a large amount of synthetic tissue fluid or deionized water. Distilled water has been used in many MTA solubility studies [7][14], however in this study, STF was selected to simulate clinical conditions due to its similarities to dentinal fluid [15]. Deionized water was also selected in the present study to keep in accordance with ISO standard evaluation process.

MTA undergoes dissolution when it is in contact with STF and releases all of its major cationic constituents and promotes the precipitation of hydroxyapatite on its surface and in the surrounding fluid [15]. Also, over long periods MTA partially releases calcium hydroxide to an aqueous environment with a decreasing rate, maintaining a high pH in aqueous solution [16].

Lower solubility was observed in the STF than deionized water group. We attribute the lower solubility of MTA in STF to the higher concentration of ions in STF than deionized water which results in lower penetration of fluid into the bulk of MTA. Also, the presence of higher concentration gradient in STF may lead to lower penetration of fluid into MTA bulk in STF group (negative gradient).

SEM micrographs revealed that bulk porosity and pore size distribution increased in specimens stored in deionized water. Secondary electron detectors revealed the presence of lumps and platelets on the surfaces of both specimens. Also, more voids were observed in specimen stored in deionized water; this finding supports the solubility results.

## CONCLUSION

The media used for evaluating solubility of a material can impact the results of study. Due to similarity of STF with biological fluids, this media is suggested as an alternative to distilled water for solubility studies of dental materials.
